# Protein activation mapping of human sun-protected epidermis after an acute dose of erythemic solar simulated light

**DOI:** 10.1038/s41698-017-0037-7

**Published:** 2017-09-21

**Authors:** Janine G. Einspahr, Clara Curiel-Lewandrowski, Valerie S. Calvert, Steven P. Stratton, David S. Alberts, James Warneke, Chengcheng Hu, Kathylynn Saboda, Elisabeth L. Wagener, Sally Dickinson, Zigang Dong, Ann M. Bode, Emanuel F. Petricoin

**Affiliations:** 1 The University of Arizona Cancer Center, 1515 North Campbell Ave., PO Box 245024, Tucson, AZ USA; 2 Department of Medicine, The University of Arizona, Tucson, AZ USA; 3 Center for Applied Proteomics and Molecular Medicine, George Mason University, Manassas, VA USA; 4 Department of Surgery, The University of Arizona, Tucson, AZ USA; 5 Department of Epidemiology and Biostatistics, The University of Arizona, Tucson, AZ USA; 6 Department of Molecular Medicine and Biopharmaceutical Sciences, The Hormel Institute, The University of Minnesota, Austin, MN USA

## Abstract

Ultraviolet radiation is an important etiologic factor in skin cancer and a better understanding of how solar stimulated light (SSL) affects signal transduction pathways in human skin which is needed in further understanding activated networks that could be targeted for skin cancer prevention. We utilized Reverse Phase Protein Microarray Analysis (RPPA), a powerful technology that allows for broad-scale and quantitative measurement of the activation/phosphorylation state of hundreds of key signaling proteins and protein pathways in sun-protected skin after an acute dose of two minimal erythema dose (MED) of SSL. RPPA analysis was used to map the altered cell signaling networks resulting from acute doses of solar simulated radiation (SSL). To that end, we exposed sun-protected skin in volunteers to acute doses of two MED of SSL and collected biopsies pre-SSL and post-SSL irradiation. Frozen biopsies were subjected to laser capture microdissection (LCM) and then assessed by RPPA. The activation/phosphorylation or total levels of 128 key signaling proteins and drug targets were selected for statistical analysis. Coordinate network-based analysis was performed on specific signaling pathways that included the PI3k/Akt/mTOR and Ras/Raf/MEK/ERK pathways. Overall, we found early and sustained activation of the PI3K-AKT-mTOR and MAPK pathways. Cell death and apoptosis-related proteins were activated at 5 and 24 h. Ultimately, expression profile patterns of phosphorylated proteins in the epidermal growth factor receptor(EGFR), AKT, mTOR, and other relevant pathways may be used to determine pharmacodynamic activity of new and selective topical chemoprevention agents administered in a test area exposed to SSL to determine drug-induced attenuation or reversal of skin carcinogenesis pathways.

## Mapping skin cancer-signaling proteins

Skin exposure to ultraviolet radiation leads to the activation of proteins involved in carcinogenic pathways. Janine Einspahr and Clara Curiel-Lewandrowski of the Arizona Cancer Center and colleagues in the US exposed normally ultraviolet protected skin of 12 individuals to two times the dose of solar-simulated light needed to induce redness. Skin biopsies were taken before and after exposure and 128 proteins known to be involved in key cancer signaling pathways were examined using ‘reverse phase protein microarray analysis’. They found early and sustained activation of multiple signaling pathways, in addition to activation of cell death and apoptosis-related proteins. The study may serve as a model for investigating the pathways involved in chronic or ultraviolet-induced carcinogenesis, which may ultimately lead to the development of targeted therapies to attenuate or reverse skin cancer pathways.

## Introduction

Skin cancer, which includes both nonmelanoma skin cancers (NMSC) and melanoma, is the most common malignancy in the United States and its incidence is rapidly increasing.^1, 2^ Skin cancer represents a significant public health and economic burden estimated to be over 8.1 billion dollars annually^3^ and consists of 75–80% basal cell carcinomas (BCC) and approximately 18% cutaneous squamous cell carcinomas (SCC). This high incidence of skin cancer can be directly related to chronic solar radiation exposure.^4^ Since the primary etiologic agent for cutaneous SCC is solar radiation, it should be possible to reduce the risk of cutaneous SCCs using current sun avoidance methods.^4^ Unfortunately, these current prevention strategies, which include sun avoidance and ultraviolet (UV) protection, have shown limited success.^5^ Therefore, there is a need to understand the molecular alterations that take place and to develop new prevention strategies to reduce the risk of skin cancers.

UV radiation is a complete carcinogen meaning that UV exposure stimulates initiation, promotion, and progression to cutaneous SCCs.^6–8^ As an initiator, UV radiation results in genetic mutations, many of which are signature UV mutations in critical genes. Additionally, UV can act as a promoter through the activation of multiple signal transduction pathways.^9^ The UV spectrum reaching the earth’s surface is 90–95% UVA (320–400 nm) and 5–10% UVB (280–320 nm).^10^ Many studies of UV-induced skin carcinogenesis have focused on UVB, which has been most closely linked to DNA damage while UVA has been associated more with skin cancer promotion. UVA and UVB have primarily been studied separately with regard to the activation of signaling pathways and skin carcinogenesis.^11–15^ Fewer studies have focused on the entire spectrum of UVA and UVB combined in a ratio that closely mimics the solar spectrum.^16^ Strong experimental evidence indicates that exposure of epidermal cells to UV results in the activation of numerous signal transduction pathways that include the PI3K/Akt-mTOR and MAPK cascades,^10^ with an effect that appears to be both time and wavelength dependent.^17^

In this study we utilized Reverse phase protein array (RPPA), a novel platform that allows for the broad-scale and quantitative measurement of the activation/phosphorylation state of dozens to hundreds of proteins to interrogate the activation of total proteins and phosphoproteins in usually sun-protected skin after an acute dose of two MED of solar stimulated light (SSL). RPPA is a powerful technology that provides a means to measure large number of ultra-low abundance signaling proteins from small amounts of input material including LCM clinical samples. Gene expression arrays are commonly utilized to measure changes in the mRNA expression, but the measurement of RNA alone could potentially miss critical information about protein drug target levels as well as post-translational modifications, such as protein phosphorylation, which drive cellular processes and control cell signaling.^18^ Pathways included the PI3k/Akt/mTOR and Ras/Raf/MEK/ERK pathways.

## Results

We exposed normally sun-protected human buttock skin to a single acute dose of two MED SSL and obtained snap frozen specimens pre- and post-SSL in 12 healthy subjects with similar types of skin (Fitzpatrick skin type II or III). The study included 8 males and 4 females [average age of 67.6 ± 10.6 (mean ± standard deviation) years for males and 66.2 ± 6.6 years for females]. All subjects were Caucasian and non-Hispanic. Fifty-eight percent (7/12) of the subjects had Fitzpatrick skin Type II (usually burns, tans less than average, with difficulty) while 42% (5/12) had skin Type III (sometimes mild burn, tans about average).^19^ The solar simulator delivered 8.7% of UVB and 91.3% of UVA^19^ and the average (±standard error) dose delivered was 3.6 ± 0.5 J/cm^2^ of UVA and 51.6 ± 7.0 mJ/cm^2^ of UVB.

Biopsies were split with ½ in formalin for immunohistochemistry (IHC)^20^ and ½ snap frozen for RPPA. Samples underwent LCM to enrich samples for epidermal cells, followed by sample lysis, and printing onto RPPA slides. RPPA slides were stained with antibodies that have been extensively pre-validated for RPPA by single band Western blotting and peptide competition. The analysis yielded data for 128 proteins/phosphoproteins, which were chosen based on literature and knowledge of signaling pathway alterations seen in both mouse and human skin after UV irradiation and in the progression of normal skin to cutaneous SCC. When the four time points post-SSL (5 min, 1, 5, and 24 h) were compared to baseline (no SSL), 42 of these investigated proteins had a *p*-value of ≤ 0.05, but due to the large number of comparisons a *p*-value of ≤ 0.0125 was required to be considered statistically significant after adjusting for multiple comparisons. This analytical process yield 33 proteins with a *p*-value of ≤ 0.0125 at any of the time points. There were four proteins with marginal significance (*p* ≤ <0.05) in any of the time points, but the Trend Test was statistically significant (*p* ≤ 0.0125). In addition, there were 15 proteins where only the Trend Test was significant (8 at *p* ≤ 0.0125 and 7 at *p* ≤ 0.05).

**Fig. 1:**
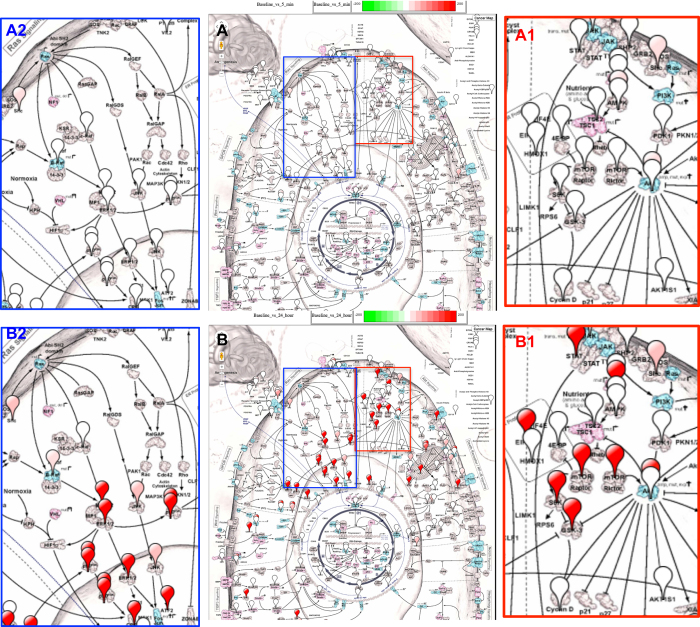
Protein pathway activation map of the AKT/mTOR and MAPK pathways. Cancer Landscape (CScape) Protein Pathway Activation Maps are shown in the center of the figure comparing the 5 min compared to baseline **a** on top and the 24 h comparison on the center bottom **b**. More significant positive differences are shown in increasing shades of red, whereas higher negative differences are shown in green. White balloons represent no significant change. Each balloon pin is placed over the protein measured. Magnified views of the Ras/Raf/MEK/ERK pathway (blue boxes, left top showing the 5 min time point, A1 and bottom right showing the 24 h time, B1) are shown to reveal pathway detail and the PI3K/AKT/mTOR pathway (red boxes, right top showing the 5 min time point, A2 and bottom left showing the 24 h time, B2). Images are modified from the “Pathways in Human Cancer” diagram courtesy of Cell Signaling, Inc

Table 1 shows the *p*-values for each time point following two MEDs of SSL (5 min, 1 h, 5 h, and 24 h) compared to baseline (no SSL). Table 1 also shows *p*-values for trend. All of the Cancer Landscape (CScape) Protein Pathway Activation Maps (example shown in Fig. 1) are available as Supplemental Figure S1). Overall, we saw activation of MAPK, AKT-mTOR and IGFR pathways, as well as the cell death/apoptosis-related pathways. Within the PI3 kinase/Akt signaling pathway, there were statistically significant results for phospho-ErbB3 (Y1289) at 1 h (*p* < 0.001) and 5 h (*p* < 0.001) when compared to baseline levels, for phospho-IRS1 (S612) at 24 h (*p* = 0.003), and for phospho-Akt (S473) at 5 h (*p* = 0.009) and 24 h (*p* = 0.006). In Fig. 1, the center, top panel compares the 5-min time point to baseline (A, top center), and the 24-hr comparison to baseline is shown in the center bottom panel (B). The protein network maps indicate that there was very little SSL-induced signaling by 5 min, whereas at 24 h, many pathways were activated. In the CScape maps, significant positive differences are demarcated (balloons) in increasing shades of red, whereas higher negative differences are shown in increasing shades of green. White balloons represent no significant change. Each balloon pin is placed over each protein that was measured. Magnified views of the PI3K/AKT/mTOR pathway (Fig. 1). Shown in the expanded CScape view are expression of phospho-mTOR (S2448), which was significantly increased at 5 h (*p* = 0.005) and 24 h (*p* < 0.001), phospho-p70 S6 Kinase (S371) with borderline significance at 5 h (*p* = 0.02) and significance at 24 h (*p* < 0.001), phospho-eIF4G (S1108) with borderline significance at 5 h (*p* = 0.02) and significant at 24 h (*p* < 0.001). phospho-GSK-3a/B (S21/9) was significantly different at 5 h (*p* = 0.002) and 24 h (*p* = 0.001), expression of p21 Waf1/Cip1 was significant at 24 h (*p* = 0.001), p27 Kip1 (*p* = 0.012) was significant at 24 h, phospho-PAK1/2 (T423/402) was significant at 24 h (*p* = 0.009), phospho-MDM2 was significantly different at (S166) at 5 h (*p* < 0.001) and 24 h (*p* < 0.001), phospho-Cyclin D1 (T286) was significant at 24 h (*p* = 0.01). Phospho-eNOS (S1177) was statistically significant at 1 h (*p* = 0.009), 5 h (*p* = 0.001), and 24 h (*p* = 0.0004), and phospho-eNOS/NOS III (S116) was significant at 24 h (*p* = 0.02). Phospho-LKB1 (S428) was significant at 5 h (*p* = 0.003) and 24 h (*p* < 0.001). Expression of phospho-MDM2 (S166) was significant at 5 h (*p* = 0.007) and at 24 h (*p* = 0.0005).

**Table 1:** *p*-values for each time point and trend following 2 MEDs of SSL

	5 min *p*-value	1 h *p*-value	5 h *p*-value	24 h *p*-value	trend test signed *p*-value
Acetylated proteins					
Acetyl-Histone H3 (Lys 9/14)	0.97	0.47	0.68	0.97	0.79
Acetyl-Histone H4 (Lys 8)	0.62	0.27	0.91	0.42	0.41
Tubulin, α acetylated	0.97	0.97	0.47	0.15	0.07
Total Proteins					
ALK	0.47	0.57	0.85	0.91	0.65
Axin1	0.85	0.38	0.97	0.092	**0.005**
Bak	0.47	0.62	0.91	0.42	0.14
Bax	0.85	0.85	0.97	0.021	0.025
Bim	0.79	0.27	0.91	0.027	**0.005**
CD24	0.52	0.52	0.85	0.42	−0.49
CDK2	0.79	0.97	0.97	0.62	0.36
Cleaved Caspase 3 (D175)	0.85	0.91	0.89	0.47	0.28
Cleaved Caspase 6 (D162)	0.91	0.38	0.79	0.021	0.028
Cleaved Caspase 7 (D198)	0.73	0.97	0.27	0.15	0.024
Cleaved Caspase 9 (D330)	0.91	0.91	0.79	0.47	0.36
Cox-2	0.52	0.97	0.62	0.021	0.017
Cyclin A	0.18	0.3	0.13	0.73	−0.54
Cyclin D1	0.18	0.23	0.91	0.85	−0.98
E-Cadherin	0.69	0.35	0.38	0.52	0.11
EGFR	0.85	0.27	0.15	0.91	0.36
ErbB2	0.68	0.97	0.73	0.62	−0.6
ErbB3	0.73	0.3	0.79	0.52	0.34
ErbB4	0.42	0.97	0.79	0.52	0.13
Estrogen Rec α	0.62	0.52	0.57	0.47	0.3
Heme-Oxygenase-1	0.73	0.34	0.68	0.23	0.15
Histone Deacetylase 4	**0.007**	0.38	0.52	0.052	−0.16
Histone Deacetylase 6	0.62	0.79	0.79	0.52	0.79
IL-10	0.96	1	0.97	0.52	0.32
MMP-11	0.68	0.79	0.91	0.18	0.039
MMP-14	0.62	0.57	0.68	0.2	0.049
Myeloperoxidase	0.68	0.3	0.15	0.42	0.18
Nanog	0.57	0.13	0.79	0.38	−0.18
Nrf2	0.91	0.97	0.38	0.34	0.3
NUMB	0.73	0.85	0.27	0.2	0.093
RANK	0.27	0.76	0.23	0.91	−0.67
RANKL	0.97	0.96	0.57	0.3	0.48
Smac/Diablo	0.97	0.23	0.15	**0.0024**	**0.00016**
Sox2	0.85	0.73	0.57	0.18	0.066
TGF-Beta	0.79	0.97	0.85	0.97	0.39
Vimentin	0.85	0.2	0.23	0.064	0.03
Wnt5a/B	0.52	1	0.79	0.57	0.35
p21 Waf1/Cip1	0.97	0.18	1	**0.0005**	**<0.0001**
p27 Kip1	0.96	0.73	0.27	**0.012**	**0.0002**
Phosphorylated Proteins					
4EBP1 (Thr 70)	0.57	0.2	0.3	0.3	0.58
Acetyl-CoA Carboxylase (Ser 79)	0.68	0.57	0.91	0.85	0.65
Adducin (Ser 662)	0.91	0.62	0.91	0.23	**0.01**
Akt (Ser 473)	0.059	0.83	**0.0092**	**0.0059**	**<0.0001**
AKT (Thr 308)	0.52	0.97	0.3	0.42	0.38
ALK (Tyr 1604)	0.27	0.3	**0.0093**	**0.0093**	**0.0002**
AMPKα1 (Ser 485)	0.064	0.52	0.97	0.39	0.31
AMPKβ1 (Ser 108)	0.97	0.42	0.91	0.15	−0.35
ASK1 (Ser 83)	0.34	0.38	0.34	0.083	0.052
ATF-2 (Thr 71)	0.79	0.022	**0.00915**	**0.0039**	**<0.0001**
ATP-Citrate Lyase (Ser 454)	0.23	0.077	**0.0049**	**0.0005**	**<0.0001**
Aurora A/B/C (Thr 288/232/198)	0.47	0.52	0.62	0.47	0.81
BAD (Ser 112)	0.48	**0.0093**	**0.00049**	**0.0005**	**<0.0001**
BAD (Ser 155)	0.79	0.052	**0.0034**	**0.0068**	**<0.0001**
Bcl-2 (Ser 70)	0.73	0.91	0.73	0.73	0.48
B-Raf (Ser 445)	0.91	0.3	0.13	0.064	**0.0033**
c-Abl (Thr 735)	0.42	0.97	0.85	0.68	−0.7
c-Abl (Tyr 245)	0.47	0.91	0.97	0.68	0.97
c-Kit (Tyr 703)	0.91	0.85	0.97	0.34	0.3
c-Kit (Tyr 719)	0.68	0.68	0.52	0.57	0.14
c-Raf (Ser 338)	0.52	0.27	0.52	0.85	0.47
CREB (Ser 133)	0.91	0.027	**0.00098**	**0.0024**	**<0.0001**
Cyclin D1 (Thr 286)	0.85	0.2	0.042	**0.012**	**0.0003**
EGFR (Tyr 1068)	0.48	0.023	**0.0039**	0.019	**0.0002**
EGFR (Tyr 1173)	0.97	0.57	0.2	0.042	**0.0015**
EGFR (Tyr 992	0.68	0.092	0.52	0.13	−0.11
eIF4G (Ser 1108)	0.56	0.27	0.016	**0.0005**	**<0.0001**
eNOS (Ser 1177)	0.85	**0.0093**	**0.00098**	**0.0005**	**<0.0001**
eNOS/NOS III (Ser 116)	0.79	0.73	0.34	0.021	**0.0027**
ErbB2 (Tyr 1248)	0.97	0.91	0.26	0.23	−0.23
ErbB2 (Tyr 877)	0.52	0.42	0.13	0.31	0.13
ErbB3 (Tyr 1289)	0.34	**0.0093**	**0.00049**	0.18	**<0.0001**
ERK1/2 (Thr 202/Tyr 204)	0.5	**0.0087**	0.034	**0.0005**	**<0.0001**
Estrogen Receptor α (Ser 118)	0.73	0.79	0.79	0.34	0.12
Ezrin/Radixin/Moesin (Thr 567/564/558)	0.52	0.34	**0.012**	0.13	**0.0041**
FADD (Ser 194)	0.022	0.058	0.032	**0.0005**	**<0.0001**
FAK (Tyr 576/577)	0.52	0.68	0.57	0.34	0.077
FKHR (Ser 256)	0.34	0.79	0.45	0.27	−0.29
Fyn (Thr 12)	1	0.68	0.62	0.34	0.14
GSK-3α/β Ser (21/9)	0.52	0.078	**0.0024**	**0.001**	**<0.0001**
Histone H3 (Ser 10)	1	0.79	0.62	0.064	0.031
IGF-1R/IR (Tyr 1135/36/1150/51)	0.31	0.1	0.26	0.18	0.14
IkBα (Ser 32/36)	0.62	0.79	0.38	0.27	0.15
IRS1 (Ser 612)	0.79	0.23	0.2	**0.0034**	**<0.0001**
Jak1 (Tyr 1022/23)	1	0.85	0.79	0.27	0.16
Lck (Tyr 505)	0.42	0.91	0.68	0.79	0.5
LIMK1/2 (Thr 508/506)	0.97	0.62	0.27	0.13	0.036
LKB1 (Ser 334)	0.91	0.2	0.15	0.52	0.12
LKB1 (Ser 428)	0.23	0.034	**0.0034**	**0.0005**	**<0.0001**
MDM2 (Ser 166)	0.73	0.052	**0.0068**	**0.0005**	**<0.0001**
MEK1/2 (Ser 217/221)	0.79	0.11	0.23	**0.0024**	**<0.0001**
Met (Tyr 1234/35)	0.97	0.97	0.38	1	0.73
Mst1/2 (Thr 183/180)	0.85	0.68	0.3	0.11	**0.01**
mTOR (Ser 2448)	0.62	0.11	**0.0049**	**0.0005**	**<0.0001**
NFkβ p65 (Ser 536)	0.91	0.91	0.27	0.97	−0.97
p38 MAPK Thr (180/Y182)	0.47	0.016	**0.0015**	**0.0024**	**<0.0001**
p70 S6 Kinase (Ser 371)	0.27	0.18	0.016	**0.0005**	**<0.0001**
p90RSK (Ser 380)	0.11	0.42	0.059	**0.0005**	**<0.0001**
p90RSK (Thr 359/S363)	0.13	0.052	**0.012**	**0.0024**	**<0.0001**
PAK1/2 (Thr 423/402)	0.79	0.57	0.13	**0.0093**	**<0.0001**
PDK1 (Ser 241)	0.91	0.52	0.68	0.23	0.099
PKA C (Thr 197)	0.91	0.62	0.68	0.18	0.069
PKCa (Ser 657)	0.91	0.47	0.68	0.27	0.11
PKCd (Thr 505)	1	0.91	0.76	0.68	0.76
PKCλζ/l (Thr 410/403)	0.73	0.97	0.47	0.79	0.72
PLCγ1 (Tyr 783)	0.52	0.016	0.3	0.91	0.59
PLK1 (Thr 210)	0.68	0.97	0.52	0.12	0.12
PRAS40 (Thr 246)	0.61	0.85	0.98	0.27	0.58
PTEN (Ser 380)	0.23	0.11	0.077	0.13	0.069
Ret (Tyr 905)	0.62	0.13	0.11	0.42	0.19
RSK3 (Thr 356/Ser 360)	0.62	0.11	**0.0093**	**0.0005**	**<0.0001**
SAPK/JNK (Thr 183/Tyr 185)	0.57	0.97	0.021	0.042	**0.0034**
SGK1 (Ser 78)	0.91	0.41	0.52	0.23	0.14
Shc (Tyr 317)	0.077	0.3	0.027	0.052	**0.0069**
SHIP1 (Tyr 1020)	0.57	0.85	0.52	0.27	0.13
SHP2 (Tyr 580)	0.85	0.73	0.57	0.52	−0.42
Smad1 (S/S)/Smad5 (S/S)/Smad8 (S/S)	0.79	0.89	0.57	0.23	0.1
SMAD2 (Ser 465/467)	0.73	0.79	0.47	0.85	0.66
Src Family (Tyr 416)	0.1	0.57	**0.0068**	0.69	0.092
Src (Tyr 527)	0.89	0.85	0.91	0.021	0.015
Stat3 (Ser 727)	0.85	0.31	0.092	**0.001**	**<0.0001**
Syk (Tyr 525/526)	0.52	0.91	0.62	0.42	−0.063
Tuberin/TSC (Tyr 1571)	0.79	0.91	1	0.42	0.54
Vav3 (Tyr 173)	0.91	0.85	0.62	0.79	0.48
VEGFR 2 (Tyr 951)	0.3	0.47	0.27	0.052	0.032
VEGFR2 (Tyr 996)	0.52	**0.0068**	**0.012**	0.79	0.049

Bolded numbers indicate the Bonferroni adjusted significance, as described in the Data and Statistical Analysis section

Within the MAPK pathways (Table 1), phospho-epidermal growth factor receptor (EGFR) (Y1068) was borderline significant at 1 h (*p* = 0.02) and statistically significant at 5 h (*p* = 0.004). Phospho-EGFR Y1173 had borderline significance at 24 h (*p* = 0.042). Figure 1 also shows CScape maps focusing on the Ras/Raf/MEK/ERK pathways [blue boxes with left top showing the 5 min time point (A1) and bottom left showing the 24 h time, (B1)]. Phospho-Shc (Y317) was borderline at 5 h (*p* = 0.03), phospho-Src Family (Y416) was statistically significant at 1 h (*p* = 0.007), and phospho-Src (Y527) had borderline significance at 24 h (*p* = 0.02). For phospho-B-Raf (S445), results were borderline at 24 h (*p* = 0.06), but the Trend Test *p*-value was significant (*p* = 0.003). Phosph-MEK1/2 (S217/221) was significant at 24 h (*p* = 0.002) while phospho-ERK1/2 (T202/Y20) was significant at 1 h (*p* = 0.009), borderline at 5 h (*p* = 0.03), and significant at 24 h (*p* = 0.005). Similar to the results for B-Raf, phospho-SAPK/JNK (T183/Y185) had borderline significance at 5 h (*p* = 0.02) and 24 h (*p* = 0.04), but the Trend Test was statistically significant (*p* = 0.003). Phospho-p38 MAPK (T180/Y182) was borderline at 1 h (*p* = 0.02) but significant at 5 h (*p* = 0.001) and 24 h (*p* = 0.002). phospho-RSK3 (T356/S360) was significant at 5 h (*p* = 0.009) and 24 h (*p* = 0.005), Phospho-p90RSK (S380) was significant at 24 h (*p* < 0.001), phospho-p90RSK (T359/S363) was significant at 5 h (*p* = 0.012) and 24 h (*p* = 0.002), phospho-CREB (S133) was borderline at 1 h (*p* = 0.03) and statistically significant at 5 h (*p* = 0.001) and 24 h (*p* = 0.002), Phospho-ATF-2 (T71) was borderline at 1 h (*p* = 0.02), while at 5 h (*p* = 0.009) and 24 h (*p* = 0.004) was statistically significant compared to baseline. Phospho-pHistone H3 (S10) showed a non-significant increase at 24 h (*p* = 0.06, Table 1).

Within the apoptotic pathway, Bim had borderline significance when compared to baseline at 24-hrs (*p* = 0.03) and had a significant Trend Test (*p* = 0.005), phospho-BAD (S112) was statically significant at 1-hr (*p* = 0.009), at 5 h (*p* = 0.005), and 24 h (*p* < 0.001). phospho-BAD (S155) was also statically significant at 5 h (*p* = 0.003) and 24-hrs (*p* = 0.007) while phospho-FADD (S194) was borderline at 5 h (*p* = 0.03) and significant at 24 h (*p* = 0.001). Phospho-ATP-Citrate Lyase (S454) was significant at 5 h (*p* = 0.0004) and at 24 h (*p* = 0.0005). Smac/Diablo was statically significant compared to baseline at 24 h (*p* = 0.002). Cleaved caspase 6 was borderline at 24 h (*p* = 0.02, Table 1)

Additional proteins showing statistically significant results were phospho-Stat 3 (S727) at 24 h (*p* < 0.001), phospho-anaplastic lymphoma kinase (ALK) (Y1604) was significant at 5 h and 24 h (*p* = 0.009). Phospho-Ezrin/Radixin/Moesin T567/564/558 was significant at 5 h (*p* = 0.012), and phospho-VEGFR2 Y996 was increased at 1 h (*p* = 0.006) and 5 h (*p* = 0.01).

To further evaluate the relevance of the 33 identified analytes demonstrating a statistically significant change from baseline to 24 h in our study (Table 1), a validity assessment was performed by comparing the change in expression with an independent cohort of subjects exposed to acute SSL. When comparing the RPPA analytes evaluated in both studies a total of 17 proteins were identified to overlap. A 94.1% agreement in directional change of expression was identified between these two cohorts, strongly supporting the validity of our findings (Table 2).

**Table 2:** RPPA analytes compared to an independent cohort

	24 h *p*-value	ssl direction	bse direction	agree/disagree
Acetylated proteins				
Smac/Diablo	0.0024	+	no match	
p2 + Waf + /Cip +	0.0005	+	+	agree
p27 Kip +	0.0 + 2	+	no match	
Phosphorylated Proteins				
Akt (Ser 473)	0.0059	+	+	agree
ALK (Tyr 1604)	0.0093	+	no match	
ATF-2 (Thr 71)	0.0039	+	no match	
ATP-Citrate Lyase (Ser 454)	0.0005	+	no match	
BAD (Ser 112)	0.0005	+	+	agree
BAD (Ser 155)	0.0068	+	+	agree
CREB (Ser 133)	0.0024	+	+	agree
Cyclin D1 (Thr 286)	0.012	+	−	disagree
eIF4G (Ser 1108)	0.0005	+	no match	
eNOS (Ser 1177)	0.0005	+	no match	
ERK1/2 (Thr 202/Tyr 204)	0.0005	+	+	agree
FADD (Ser 194)	0.0005	+	no match	
GSK-3α/β Ser (21/9)	0.001	+	+	agree
IRS1 (Ser 612)	0.0034	+	no match	
LKB1 (Ser 428)	0.0005	+	+	agree
MDM2 (Ser 166)	0.0005	+	+	agree
MEK1/2 (Ser 217/221)	0.0024	+	+	agree
mTOR (Ser 2448)	0.0005	+	no match	
p38 MAPK Thr (180/Y182)	0.0024	+	+	agree
p70 S6 Kinase (Ser 371)	0.0005	+	+	agree
p90RSK (Ser 380)	0.0005	+	+	agree
p90RSK (Thr 359/S363)	0.0024	+	+	agree
PAK1/2 (Thr 423/402)	0.0093	+	+	agree
RSK3 (Thr 356/Ser 360)	0.0005	+	no match	
Stat3 (Ser 727)	0.001	+	+	agree

The listed proteins correspond to the 33 analytes demonstrating a significant change from baseline to 24 h. These proteins were compared with an independent cohort of subjects acutely exposed to 2MED of SSL. A total of 17 proteins overlapped between both studies. A similar direction in expression change was identified in 16/17 of the measured analytes indicating a 94.1% agreement between both studies

## Discussion

In the current study, we utilized RPPA, a powerful proteomics platform that provides for the broad-scale and quantitative measurement of the activation/phosphorylation state of dozens to hundreds of proteins at once from a single input sample. We used RPPA to interrogate the activation of more than 100 specific total proteins and phosphoproteins that comprised key signaling “hubs” in known cancer pathways that are causally involved in growth and mitogenesis, survival, motility, apoptosis, autophagy, inflammation and energy metabolism in sun-protected skin after an acute dose of two MED of SSL. We are not aware of any other studies using the RPPA platform for the broad-scale and quantitative measurement of proteins and phosphoproteins in SSL-irradiated skin. While gene expression arrays are commonly utilized to measure changes in the mRNA expression, the measurement of RNA alone lacks critical information such as post-translational modifications such as protein phosphorylation which drive cellular processes and control cell signaling.^18^

While an important novel attribute of our study is the evaluation of acute SSL effects on human subjects in a clinical setting, a key limitation of RPPA is that we are limited by the size and location of the clinical biopsy sample and thus we cannot perform “kinome wide” measurements of every cellular signalling protein as one could do with billions of cells grown in culture. The number of cells that could be captured by LCM of epidermis was ~15,000 cells, and thus reducing the number of RPPA proteins that we were able to analyse and present here. Consequently, we chose to focus our analysis on key signalling pathways involved in the epidermal UV-response and in tumorigenic processes such as stress, inflammation, survival, energy metabolism, growth, and differentiation. Our protein panel was based on the literature and knowledge of signaling pathway alterations seen in both mouse and human skin after UV irradiation and in the progression of normal skin to cutaneous SCC. Furthermore, while signalling can be regulated by a number of post-translational modification driven events (e.g., glycosylation, acetylation, etc.), we elected to focus on protein phosphorylation, which commonly drives signalling cascades of interest. As for most immunoassays, RPPA requires the availability of well-performing, highly specific antibodies. Our study included 128 proteins to determine whether an acute dose of two MED of SSL to normally sun-protected skin of healthy volunteers would activate the expression of key cell signaling pathways that are also activated or are altered in expression during the progression of normal skin, to sun-damaged skin, to AK, and finally to SCC. Of the 128 key signaling proteins measured, only a subset of activated proteins achieved statistical significance after correcting for multiple comparisons (Table 1).

**Fig. 2:**
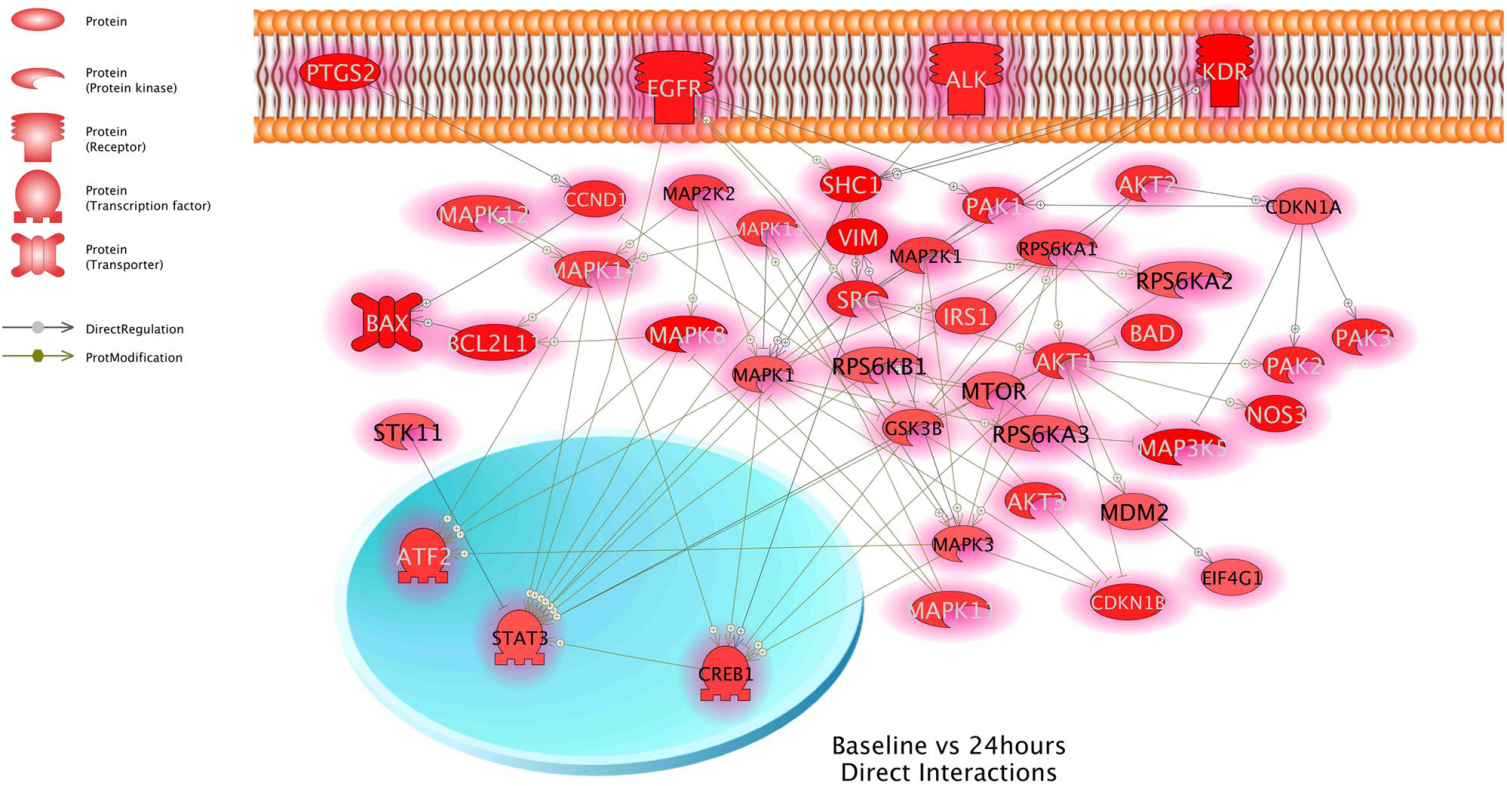
Protein network analysis of two MED SSL effects on normal sun protected skin at 24 h. Protein network analysis was also conducted using the statistically significant proteins from the RPPA data. Only the statistically significant proteins and phosphoproteins within each time point were used for this analysis

In our study, 6 mm skin biopsies from a sun-protected site were split in half with one half fixed in formalin for IHC (published in Bermudez et al^20^) and the other half were snap frozen for RPPA. We found evidence of biochemically linked activation of multiple signaling pathways that included a number of individual members of the MAPK pathway, the AKT-mTOR signaling pathways and the cell death/apoptosis-related pathways as well as specific RTK activation such as EGFR, ALK, and vascular endothelium growth factor receptor (VEGFR). There was early and sustained activation of the p38/SAPK/JNK/ERK pathways beginning at 1 h [i.e., p38 (T180/Y182), ERK1/2 (T202/Y204), MEK1/2 (S217/221), CREB (S133), RSK3 (T356/S360)], while at 5 h expression of these phosphoproteins/proteins was increased for both the number of proteins expressed and the levels of expression [i.e., SAPK/JNK (T183/Y185), ATF-2 (T71), p90RSK (T359/S363)]. Furthermore, the levels of expression were sustained or even further increased at 24 h. Similarly, activation of the AKT-mTOR pathways was generally increased beginning at the 1-hr time point with modest additional increases in phosphorylation of GSK3α/β (S21/9) and p70 S6 Kinase (S371). Furthermore, at 5 and 24 h phosphorylation of AKT (S473) and mTOR (S2448) were statistically significantly increased. Phospho-eIF4G (S1108) was significantly increased at only 24 h. With the exception of FADD (S194), apoptotic proteins were not evident at 5 min, but at 1 h, ATP-Citrate Lyase (S454), BAD (S112), and BAD (S155) become evident at 5 and 24 h. ATP-Citrate Lyase (S454), BAD (S112), and BAD (S155) were further increased. Some additional proteins become evident and others increased at 24 h (Cleaved Caspase 6, Smac/Diablo, and Bax). We used two independent pathway visualization and network connectivity tools (Figs. 1 and 2), both of which pointed to systemic pathway-based signaling activation of the MEK-ERK, AKT-mTOR, and selected RTK activation.

In a recent publication,^20^ we utilized the formalin-fixed portion of the biopsy from our current study to determine whether SSL would activate the expression of key proteins/phosphoproteins within the PI3K/Akt/mTOR and MAPK signaling pathways using IHC. Our IHC assays are quantified using image analysis to give a result as the percent positive area of the epidermis to yield data as a continuous variable. Findings from these IHC analyses generally support our RPPA findings herein. Results included a significant but modest increase in AKT (S473) at 5 h and a significant and sustained increase in mTOR (S2448) over 24 h. We did not measure the same phosphorylation site of 4E-BP1 (T37/46) between IHC and RPPA but found a significant increase at 24 h through both methodologies. Phospho-p38 (T180/Y182) peaked between 1 and 5 h, and this level was sustained up to 24 h. Expression of phospho-ERK1/2 (T202/Y204) was evident at 5 min and was then increased and sustained to 24 h. Several proteins like COX-2 and cleaved caspase three were significantly increased by IHC; although in some cases RPPA also indicated that levels were increased, the difference was not statistically significant. In another study from our group in which we used four MED of UV in 23 volunteers, we found activation of proteins within the MAPK, PI-3K, p53 and JNK pathways via IHC.^15^ At four MED, we also found significant changes in epidermal expression of phospho-CREB and phospho-GSK-3β from 30 min to 24 h. The power to detect the change in any expression level from baseline to 24 h post exposure depends on both the strength of the effect and also on the total number of tests performed (which is equal to the total number of markers/analytes being tested). Since the RPPA analysis considers a much larger number of analytes simultaneously compared to the IHC analysis, the power of the two approaches cannot be directly compared.

Since skin is exposed daily to low dose solar radiation, we believe that the investigation of signaling pathway alterations that result from acute exposures may be relevant to those changes that are seen during skin carcinogenesis. UV is responsible for both initiation and promotion phases of carcinogenesis (a “complete” carcinogen), and chronic exposure to UV results in an accumulation of the critical gene mutations required to drive cells to malignancy; but UV can also act during the promotion phase of carcinogenesis, which could manifest as altered cell signaling ultimately leading to the development of SCC. Interestingly, Martincorena et al.^21^ recently reported that in normal aged sun-exposed eyelid skin there were multiple cancer genes under strong positive selection, including most of the key drivers of cutaneous SCC. They concluded that aged sun-exposed skin was in reality a patchwork of thousands of evolving clones, and while a significant number of these clones contained cancer-causing mutations, the tissue but was able to maintain normal histology.

Using RPPA, we previously^22^ found statistically significant differences in protein expression and activation within the MEK-ERKs and Akt/mTOR signaling pathways in AK compared to SCC and normal skin.^22^ Of interest to the current study, we found that the phosphorylated forms of p38 (T180/Y182), ERK1/2 (T202/Y204), MEK1/2 (S217/221), mTOR (S2448), eIF4G (S1108), p70S6K1 (S371), 4EBP1 (T70), and GSK-3α/β (S21/9), were significantly increased in SCC compared to AK. Furthermore, phospho-4EBP1 (S65) and phospho-Akt (S473) were increased in AK compared to normal skin.^22, 23^ Our broader pathway activation mapping analysis performed and described herein provide the most comprehensive signaling analysis ever performed for UV exposure effects. Our analysis confirmed these previous findings^23^ and also implicated activation of other signaling architecture such as receptor tyrosine kinases EGFR, ALK, and VEGFR amongst other proteins described in Table 1.

IHC and RPPA provide complementary assessment of proteins involved in pathways of interest and validation on results when endpoints are shared across both methodologies. In our current study, samples for RPPA were subjected to LCM to highly enrich for the epithelial cells of interest. However, it is still not possible to determine which cells within the epidermis are expressing a particular protein because cells are lysed. In contrast, in situ measurement of proteins by IHC and image analysis can be fairly easily ascertained in most fixed samples.

We propose that the study of the acute effects of UV on normal skin may serve as a model for the investigation of the effects of SSL on the complex array of signal transduction pathways. It is likely that at least some of these pathways are involved in chronic or UV-induced carcinogenesis. The study of both acute and chronic solar light exposure may also serve as a model whereby critical signaling pathways and individual proteins can be identified as potential biomarkers for use as endpoints in clinical trials or as companion biomarkers. Moreover, the study of both acute and chronic solar light exposure may allow for the development of interventions such as targeted therapies in a much shorter amount of time than required for studies using cancer as an endpoint.

## Materials and methods

### Study population

Study participants were recruited from a pool of subjects who had been previously screened and/or participated in previous skin chemoprevention trials and had agreed to be re-contacted for future studies. The eligibility criteria for participants from this pool included age of 18 years or older and Fitzpatrick skin types II (burns easily, tans poorly) or III (burns moderately, tans gradually). Exclusion criteria included immunosuppression, serious concurrent illness, invasive cancer (including any type of skin cancer) within the past 5 years and baseline serum chemistry values outside of normal limits. In addition, those using photosensitizing medications or topical medications on the test area during the past 30 days were ineligible. Individuals taking mega doses of vitamins were not eligible (i.e., more than five times the RDA, more than five capsules of multivitamins, 400 IU of vitamin E, 200 g of selenium, and 1 g of vitamin C). Additional exclusion criteria included individuals with a history of sun exposure to the buttocks within 30 days of randomization and participants must have agreed to avoid sun exposure during the study period. Finally, individuals with a known allergy to lidocaine were ineligible. The University of Arizona Institutional Review Board approved the study and written informed consent was obtained from all study participants. Methods were performed in accordance with relevant regulations and guidelines.

### Administration of solar simulated light

#### Minimal erythema dose (MED)

The MED of SSL was determined for each individual using a Multiport UV Solar Simulator Model 600 (Solar Light Co., Philadelphia, PA, USA) and corroborated by using a reflectance spectrophotometer (Minolta Chroma Meter Model CR-200; Minolta Corporation, Osaka, Japan). The spectrum of light generated by the Solar Simulator consisted of 8.7% UVB and 91.3% UVA.^19^ The dose of emission was precisely regulated to be limited to UVA and UVB spectra (290–390 nm). MED was defined as the smallest dose of energy necessary to produce confluent erythema with four distinct borders at 24 h post-exposure. MED was determined on a buttock area previously unexposed to sunlight. Each test area was subdivided into six subsites (each 1 cm^2^) corresponding to the liquid light guide pattern on the solar simulator. The solar simulator was calibrated prior to each use and a series of six increasing SSL radiation exposures were administered to each subsite area. Following exposure, the test sites were covered until evaluations were completed.^20^

#### Administration of 2X MED

After determination of the MED for each individual, the contralateral buttock was exposed to 2 times the MED. A 4 mm skin punch biopsy sample was collected from one buttock at baseline prior to SSL exposure and additional 4 mm punch biopsies were removed at 5 min, 1, 5, and 24 h post-SSL irradiation. Biopsy sites were then sutured and subjects returned to the clinic for suture removal at ~1 week.

### Reverse phase protein array

Highly enriched populations of ~6000 epithelial cells were obtained from skin samples by UV cutting laser microdissection using the Veritas (Molecular Devices, Sunnyvale, CA, USA). Microdissected cells were lysed on the CapSure^®^ Macro LCM Caps (Molecular Devices, Sunnyvale, CA, USA) using with a 1:1 mixture of T-Per™ Tissue Protein Extraction Reagent (Pierce, Rockford, IL, USA) and 2X Tris-Glycine SDS Sample Buffer (Invitrogen, Carlsbad, CA, USA) containing 5% ß-mercaptoethanol. Reverse Phase Protein Microarrays were prepared using an Aushon 2470 solid pin microarrayer (Aushon Biosystems, Billerca, MA, USA). A series of positive control lysates consisting of cell lines treated with compounds that cause broad phosphoprotein increases (e.g., pervanadate, calyculin) or negative controls (untreated cells) were also printed and slides were stored desiccated at −20 °C prior to staining with antibody. For estimation of total protein amounts, selected arrays were stained with Sypro Ruby Protein Blot Stain (Invitrogen, Carlsbad, CA, USA) according to the manufacturer’s instructions and visualized on the NovaRay scanner (Alpha Innotech, San Leandro, CA, USA). Printed slides were prepared for staining by treating with 1x Reblot (Chemicon, Temecula, CA, USA) for 15 min, followed by 2 × 5 min washes with PBS. Slides were treated for at least 5 h or overnight with blocking solution (1 g I-block; Applied Biosystems, Bedford, MA, USA), 0.5% Tween-20 in 500 mL PBS) with constant rocking. Blocked arrays were stained with antibodies on an automated slide stainer (Dako North America, Inc., Carpinteria, CA, USA) using the Catalyzed Signal Amplification System kit according to the manufacturer’s recommendation (CSA; Dako). Briefly, endogenous biotin was blocked for 10 min with the biotin blocking kit (Dako), followed by application of protein block for 5 min; primary antibodies were diluted in antibody diluent and incubated on slides for 30 min and biotinylated secondary antibodies were incubated for 15 min. Signal amplification involved incubation with a streptavidin-biotin-peroxidase complex provided in the CSA kit for 15 min, and amplification reagent (biotinyl-tyramide/hydrogen peroxide, streptavidin-peroxidase) for 15 min each. A signal was generated using streptavidin-conjugated IRDye680 (LI-COR Biosciences, Lincoln, NE, USA). Slides were allowed to air dry following development. All antibodies were subjected to extensive validation for single band, appropriate MW specificity by Western blot, as well as phosphorylation specificity through the use of cell lysate controls (e.g,. HeLa cells +/− pervanadate, Jurkat cells +/− Calyculin purchased from Cell Signaling as lysates). The RPPA community has developed best practices and conventions for antibody validation.^24^ These include single band validation on Western blot, as well as the use of positive and negative controls that are printed on the same array as the experimental samples. These methods have been used by our lab and reported in over 150 peer-reviewed publications to date. Indeed, in early publications when key RPPA findings were evaluated by Western blot, all findings were successfully validated.^25^ Importantly, RPPA findings have also been cross-validated using FDA-approved means of measuring the same protein such as parallel IHC and FISH evaluation of HER2 expression.^26^ In this publication, the RPPA findings showed 95 and 97% concordance with IHC Herceptest™ and FISH HER2 assays. A full list of all antibodies used in this study along with the vendor and dilution used are shown (Supplemental Table 1).

Stained slides were scanned individually on the NovaRay scanner (Alpha Innotech) or the Vidar Scanner (Vidar Systems, Herndon, VA). The TIF images for antibody-stained slides and Sypro-stained slide images were analyzed using MicroVigene v2.9.9.9 (VigeneTech, Carlisle, MA, USA). Briefly, Microvigene performed spot finding, local background subtraction, replicate averaging and total protein normalization, producing a single value for each sample at each endpoint. All data was background subtracted (local and slide average), normalized to total protein, and all signal values produced for data analysis were at least two standard deviations above background.

### Data and statistical analysis

A total of 128 RPPA proteins were included in the analysis. Each protein was measured at five time points: baseline (BL), 5 min, 1 h, 5 h, and 24 h after exposure to SSL. Supplemental Table 2 contains raw RPPA data for each subject and time point. The distribution of each protein was first examined graphically using histograms and box plots, and the appropriate transformation applied, if necessary, to achieve approximate normality. An overall homogeneity test was first performed to check whether the levels of each protein at the different time points were the same. This test was implemented by fitting a generalized estimating equations (GEE) model for protein values across all time points and performing a multivariate Wald test on estimated parameters from this model. GEE was used to account for within-subject correlation. Due to the large number of proteins evaluated, the Benjamini–Hochberg method was used to adjust for multiple testing with the false discovery rate controlled at the 0.05 level. If the overall homogeneity test for any protein remained significant after the multiple testing adjustment (indicating that the levels of the protein were not the same across the five time points). The Wilcoxon signed-rank test was then conducted for each protein to compare the level at baseline to the levels at 5 min, 1 h, 5 h, and 24 h after SSL exposure. Since four pair-wise comparisons were made for each protein, the Bonferroni method was used to adjust for multiple comparisons, with significance level set at 0.05 divided by the four timepoints (*p* ≤ 0.0125). In addition, a trend test was also conducted to check whether there was a monotonic trend from baseline to 24 h after SSL exposure. For this test, a GEE model was fitted for levels of each protein with time points as covariate (coded as numerical values 0, 1, 2, 3, and 4). As previously indicated, due to the large number of proteins, the Benjamini–Hochberg method was used to adjust for multiple testing, with the false discovery rate controlled at the 0.05 level. We also validated our findings using an independent validation set. For each analyte with significant change (*p* < 0.0125) from baseline to 24 h, analyte in the validation study representing the same proteins is identified, if existent, and the direction of change from baseline to 24 h post-exposure was compared to the direction of change in this study. The rate of agreement was then estimated.

### Pathway mapping visualization and protein network analysis

Statistically significant data derived from the RPPA analysis was visualized for network linkages as previously described.^27^ Briefly, the “Pathways in Human Cancer” diagram (reproduced courtesy of Cell Signaling, Inc., www.cellsignal.com) along with the Google Maps Application Programming Interface (API) (http://code.google.com/apis/maps) was used to create a dynamic web application called CScape (Cancer Landscape) for visualizing and navigating through the RPPA-generated data.

Protein network analysis was also conducted using the statistically significant proteins from the RPPA analysis [Pathway Studio v.11.0 (Elsevier, Amsterdam, Netherlands)]. The software contains the ResNet Mammal v. 11.0 database with functional relationships and pathways of human, mouse and rat genes derived from over 24,000,000 PubMed abstracts and over 3500000 papers from Elsevier and 3rd party groups. The list of significant proteins and phosphoproteins was first mapped to the Huge Gene Nomenclature Committee (HGNC)-approved gene symbols, which were inputted into the software. For significant proteins and phosphoproteins within each time point, we conducted pathway enrichment analysis based on the Fisher’s exact test and a *p* value cut off of 0.05. We then generated interaction networks to determine the direct interactions between the significant proteins/phosphoproteins. Each interaction in the network is supported by at least one published reference from the ResNet Mammal v.11.0 database. Figure 2 shows one of these networks used to visualize the RPPA data from human skin biopsies obtained 24 h post-SSL. Statistical analysis found a series of SSL-induced phosphoprotein and protein activation/expression changes that occurred at 24 h. The results showed significant activation of a number of receptor tyrosine kinases (EGFR, ALK, VEGFR) along with downstream COX2 signaling, mitogenic (MEK-ERK pathway), pro-survival (AKT-mTOR pathway) and apoptosis pathways (Fig. 2)

### Data availability statement

The raw data that support the findings of this study is included Supplemental Table 2. Upon reasonable request further information can be requested from the University of Arizona Prevention of Skin Cancer P01 Data Sharing Committee (UACC-SkinPPG@uacc.arizona.edu).

## Electronic supplementary material

Supplemental Figure 1

Supplemental Table 1

Supplemental Table 2

## Electronic supplementary material

**Supplementary Information** accompanies the paper on the *npj Precision Oncology* website (doi:10.1038/s41698-017-0037-7).
