# Supplementary material for: Protein activation mapping of human sun-protected epidermis after an acute dose of erythemic solar simulated light
Source: NPJ Precis Oncol. 2017 Sep 21;1:34. doi: 10.1038/s41698-017-0037-7 (PMC5695572; doi:10.1038/s41698-017-0037-7)
Supplement: Supplementary file 1 — Supplemental Figure 1 [file 41698_2017_37_MOESM1_ESM.pdf]

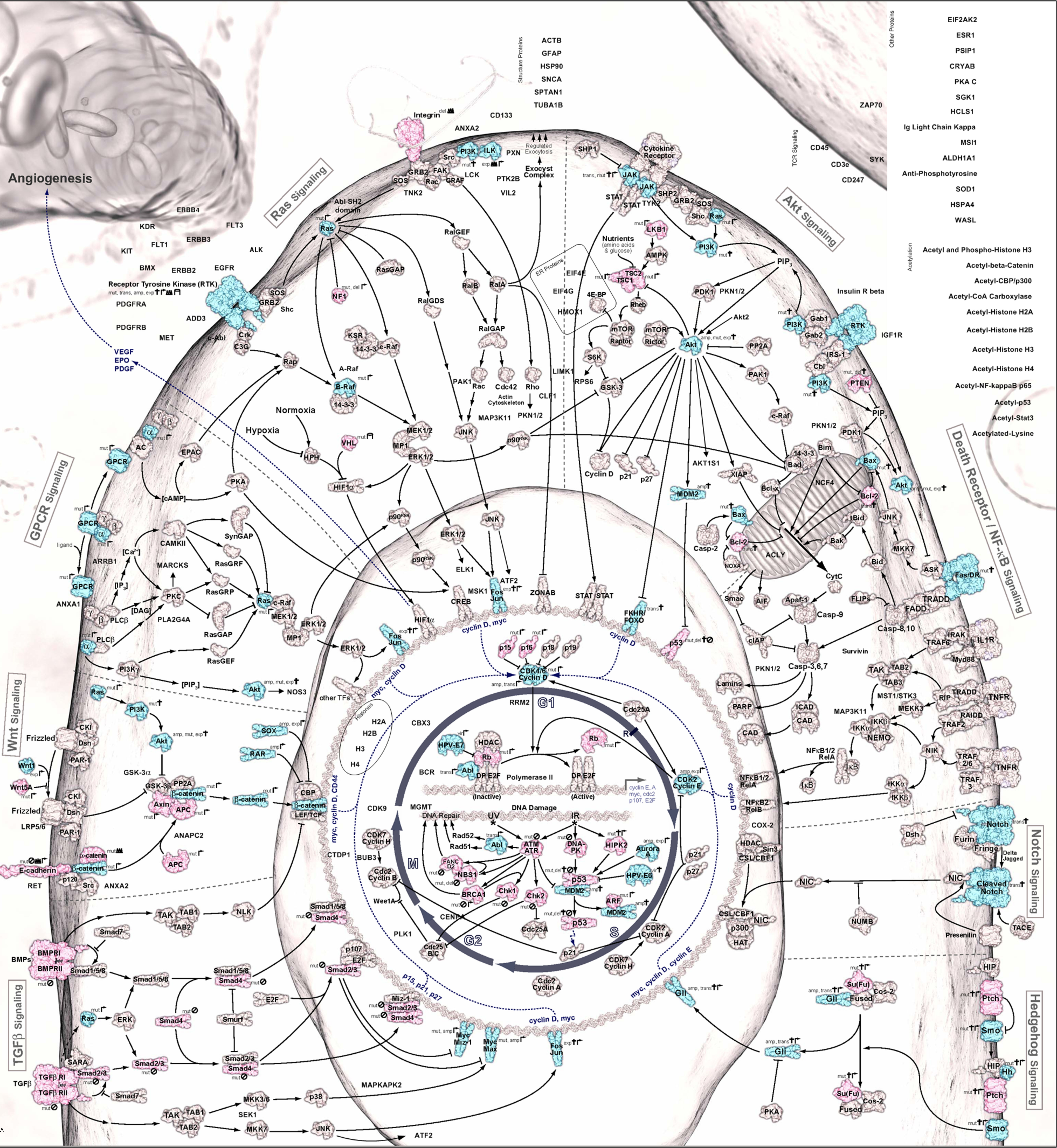

Angiogenesis
Ras Signaling
Akt Signaling
GPCR Signaling
Wnt Signaling
TGFβ Signaling
Death Receptor / NF-κB Signaling
Notch Signaling
Hedgehog Signaling
Structure Proteins
ACTB
GFAP
HSP90
SNCA
SPTAN1
TUBA1B
Other Proteins
EIF2AK2
ESR1
PSIP1
CRYAB
PKA C
SGK1
HCLS1
Ig Light Chain Kappa
MSI1
ALDH1A1
Anti-Phosphotyrosine
SOD1
HSPA4
WASL
TCR Signaling
ZAP70
CD45
CD3e
SYK
CD247
Acetylation
Acetyl and Phospho-Histone H3
Acetyl-beta-Catenin
Acetyl-CBP/p300
Acetyl-CoA Carboxylase
Acetyl-Histone H2A
Acetyl-Histone H2B
Acetyl-Histone H3
Acetyl-Histone H4
Acetyl-NF-kappaB p65
Acetyl-p53
Acetyl-Stat3
Acetylated-Lysine
Integrin
CD133
ANXA2
Regulated Exocytosis
Exocyst Complex
PXN
PTK2B
VIL2
PI3K
ILK
Src
FAK
GRB2
SOS
Rac
GRAF
LCK
TNK2
Abl SH2 domain
Cytokine Receptor
SHP1
JAK
STAT
TYK2
SHP2
Shc
Ras
KDR
ERBB4
FLT3
FLT1
ERBB3
KIT
BMX
ERBB2
EGFR
ALK
Receptor Tyrosine Kinase (RTK)
PDGFRA
PDGFRB
ADD3
MET
c-Abl
Crk
C3G
VEGF
EPO
PDGF
RalGEF
RasGAP
NF1
RalGDS
RalB
RalA
RalGAP
KSR
14-3-3
c-Raf
A-Raf
B-Raf
Rap
PAK1
Cdc42
Rho
Actin Cytoskeleton
MAP3K11
PKN1/2
CLF1
Normoxia
Hypoxia
HPH
VHL
HIF1α
MEK1/2
MP1
ERK1/2
p90RSK
JNK
ER Proteins
EIF4E
EIF4G
HMOX1
4E-BP
Nutrients (amino acids & glucose)
LKB1
AMPK
TSC2
TSC1
Rheb
mTOR
Raptor
Rictor
S6K
RPS6
GSK-3
LIMK1
PIP3
PDK1
Akt2
Akt
PP2A
Cyclin D
p21
p27
AKT1S1
XIAP
MDM2
Insulin R beta
Gab1
Gab2
RTK
IGF1R
IRS-1
Cbl
PTEN
Bad
Bim
Bax
NCF4
Bcl-xL
Bcl-2
tBid
Bak
Casp-2
NOXA
ACLY
CytC
Smac
AIF
Apaf-1
Casp-9
cIAP
Casp-3,6,7
Survivin
Bid
MKK7
ASK
Fas/DR
FLIPS
FADD
TRADD
Casp-8,10
IRAK
TRAF6
IL1R
Myd88
TAK
TAB2
TAB3
MST1/STK3
MEKK3
RIP
TNFR
RAIDD
TRAF2
NIK
IKKα
IKKβ
NEMO
IκB
NFκB1/2 RelA
NFκB2 RelB
TRAF 2/6
TRAF 3
Lamins
PARP
ICAD
CAD
COX-2
Dsh
Notch
Furin
Fringe
Delta Jagged
NIC
Cleaved Notch
NUMB
TACE
Presenilin
CSL/CBF1
p300
HAT
HDAC
Sin3
HIP
Ptch
Smo
Hh
Su(Fu)
Gli
Fused
Cos-2
PKA
GPCR
AC
EPAC
PKA
[cAMP]
ligand
ARRB1
[Ca2+]
[IP3]
[DAG]
CAMKII
MARCKS
SynGAP
RasGRF
RasGRP
RasGEF
PKC
PLCβ
PLA2G4A
ANXA1
CREB
MSK1
ELK1
Fos
Jun
ATF2
ZONAB
STAT STAT
FKHR/FOXO
NOS3
Frizzled
Wnt1
Wnt5A
CKI
PAR-1
LRP5/6
GSK-3α
GSK-3β
Axin
APC
β-catenin
ANAPC2
α-catenin
E-cadherin
RET
p120
CBP
LEF/TCF
SOX
RAR
other TFs
Histones
H2A
H2B
H3
H4
cyclin D, myc
myc, cyclin D
myc, cyclin D, CD44
p15
p16
p18
p19
CDK4/6 Cyclin D
RRM2
G1
CBX3
HPV-E7
Rb
DP E2F
BCR
Abl
Polymerase II
(Inactive)
(Active)
cyclin E, A myc, cdc2 p107, E2F
Cdc25A
CDK2 Cyclin E
R
MGMT
DNA Damage
DNA Repair
UV
IR
CDK9
CDK7 Cyclin H
BUB3
Cdc2 Cyclin B
Wee1A
Rad52
Rad51
ATM ATR
DNA-PK
HIPK2
Aurora A
FANC D2
NBS1
BRCA1
Chk1
Chk2
p53
HPV-E6
ARF
CENPA
Cdc25A
Cdc25 B/C
PLK1
M
G2
S
CDK2 Cyclin A
Cdc2 Cyclin A
CTDP1
p21
p27
cyclin D
myc, cyclin D, cyclin E
BMPs
BMPRI
BMPRII
Smad1/5/8
Smad7
Smad4
Smad2/3
Smurf
ERK
TAB1
NLK
SARA
TGFβ
TGFβ RI
TGFβ RII
p107
E2F
Miz-1
Myc
Max
p15, p21, p27
MAPKAPK2
MKK3/6
p38
SEK1
MKK7
mut
amp
trans
exp
del

A

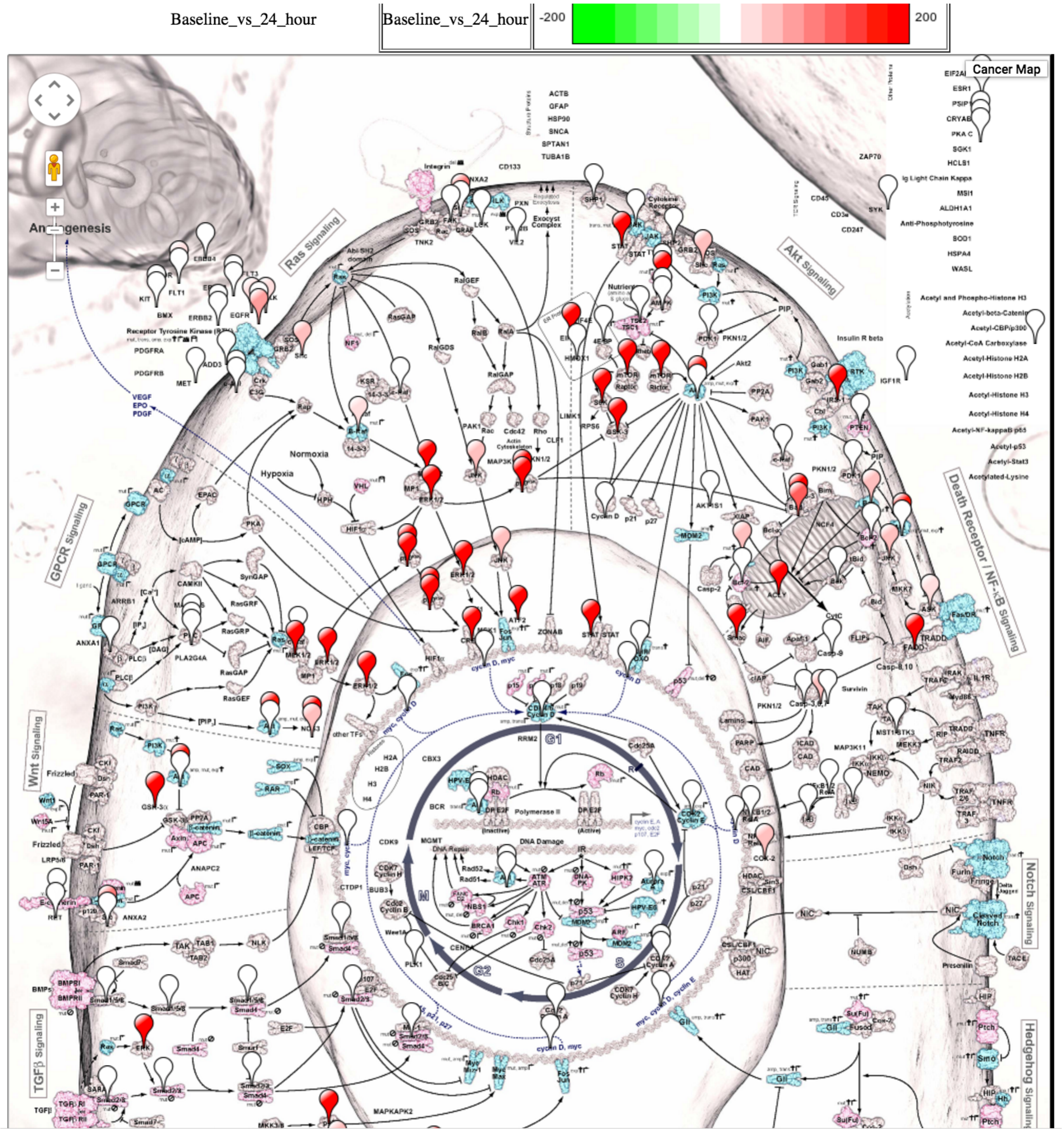

Baseline_vs_24_hour
Baseline_vs_24_hour
-200
200
Cancer Map
Angiogenesis
Ras Signaling
Akt Signaling
GPCR Signaling
Wnt Signaling
TGFβ Signaling
Death Receptor / NF-κB Signaling
Notch Signaling
Hedgehog Signaling
ACTB
GFAP
HSP90
SNCA
SPTAN1
TUBA1B
CD133
Integrin
Exocyst Complex
ZAP70
CD45
CD3e
CD247
SYK
ESR1
CRYAB
SGK1
HCLS1
Ig Light Chain Kappa
MSI1
ALDH1A1
Anti-Phosphotyrosine
SOD1
HSPA4
WASL
Acetyl and Phospho-Histone H3
Acetyl-beta-Catenin
Acetyl-CBP/p300
Acetyl-CoA Carboxylase
Acetyl-Histone H2A
Acetyl-Histone H2B
Acetyl-Histone H3
Acetyl-Histone H4
Acetyl-NF-kappaB p65
Acetyl-p53
Acetyl-Stat3
Acetylated-Lysine
Receptor Tyrosine Kinase (RTK)
PDGFRA
PDGFRB
KIT
FLT1
BMX
ERBB2
EGFR
MET
ADD3
VEGF
EPO
PDGF
Insulin R beta
IGF1R
Normoxia
Hypoxia
Cyclin D
p21
p27
G1
M
G2
S
Polymerase II
DNA Damage
DNA Repair
RRM2
CBX3
BCR
CDK9
MGMT
CTDP1
BUB3
PLK1
MAPKAPK2
PKN1/2
Survivin
MAP3K11
NEMO

B

Supplemental Figure 1. High resolution protein pathway activation map. Cancer Landscape (CScape) Protein Pathway Activation Maps are shown comparing the baseline (A) and the 24 hours comparison (B). More significant positive differences are shown in increasing shades of red, whereas higher negative differences are shown in green. White balloons represent no significant change. Each balloon pin is placed over the protein measured. Images are modified from the "Pathways in Human Cancer" diagram courtesy of Cell Signaling, Inc.
